# The Efficacy of Giant Phikz Viruses in Degrading Biofilms of *Pseudomonas aeruginosa* in Comparison to Pbuna Viruses

**DOI:** 10.3390/v18080869

**Published:** 2026-08-09

**Authors:** Yueqi Wang, Maria Bourkaltseva, Alexander Burykin, Sergey Krylov, Elena Pleteneva, Victor Krylov, Olga S. Sokolova

**Affiliations:** 1 Faculty of Biology, Shenzhen MSU-BIT University, 1 International University Park Road, Dayun New Town, Longgang District, Shenzhen 518172, China; 3120210002@smbu.edu.cn; 2 Faculty of Biology, Lomonosov Moscow State University, Leninskie Gory 1, Bld 12, Moscow 119234, Russia; 3 Mechnikov Research Institute of Vaccines & Sera, Maly Kazenny Side-Street 5a, Moscow 105064, Russia; mariabour@mail.ru (M.B.); alextalion39@gmail.com (A.B.); murk505@yahoo.com (S.K.); epletmech@yandex.ru (E.P.); krylov.mech.inst@mail.ru (V.K.); 4 Lomonosov Institute of Fine Chemical Technologies, MIREA–Russian Technological University, Moscow 119571, Russia

**Keywords:** biofilms, clinical isolates, *Pseudomonas aeruginosa*, bacteriophages, exopolysaccharides, SEM, phage therapy

## Abstract

*Pseudomonas aeruginosa* poses major public health threats, due to its robust, treatment-resistant biofilms, which contribute to multi-drug resistance. Bacteriophages offer a promising alternative. This study evaluates giant Phikzvirus and conventional Pbunavirus phages against pre-formed biofilms from four multi-drug-resistant *P. aeruginosa* clinical isolates. We tested six phages (three Phikzvirus, three Pbunavirus) against four clinical strains, isolated from chronic urological and pulmonary infections (Ur1, Ur14, Lu3, Lu9) at MOIs 0.001–0.1, quantifying biofilm biomass by crystal violet and visualizing architecture by SEM. Phage treatment leads to significant disrupted biofilms in three isolates achieving more than 50% reduction—comparable with typical antibiotic efficacy against mature biofilms. All biofilms retained their EPS architecture after the treatment, as revealed by SEM, suggesting that residual eDNA-polysaccharide complexes could maintain structural cohesion even after bacterial lysis. The phage’s ability to reduce biofilm biomass demonstrated a significant dependence on the multiplicity of infection (MOI). The best results of anti-biofilm activity of phages (reduction in biofilm biomass by more than 75%) were observed for the phage phiKZ for two strains—Ur1 at MOI = 0.001 and Lu9 at MOI = 0.01—and for the phage phi14/1 for the Ur1 strain at MOI = 0.001. The biofilm formed by the antibiotic-resistant clinical isolate Ur14 demonstrated exceptional resistance to both types of bacteriophages despite the sensitivity of bacteria of this strain to the studied phages. This highlights the need for personalized phage therapy, where tailored phage cocktails are selected for each specific bacterial strain to achieve optimal destruction of the biofilm.

## 1. Introduction

*Pseudomonas aeruginosa* is a Gram-negative bacterium that causes a wide range of hospital-acquired infections [1,2,3], due to the inherently high and rapidly acquired resistance of clinical strains to most antibiotics [4,5]. Importantly, *P. aeruginosa* can form complex organized communities—biofilms, protecting themselves from various stressors, including drugs and host defenses. In industrial applications, biofilms cause machine failures, equipment corrosion, clogged pipelines, contamination of drinking water distribution systems, and safety problems in the food industry [6]. In hospitals, biofilms are associated with the use of medical implanted equipment—lenses, prostheses, and artificial heart valves [7]. They may also lead to urinary catheter-associated infection and surgical/transplant infections [1]. Specifically, *P. aeruginosa* can most often infect central venous and urinary catheters, as well as artificial hip prostheses [8]. Biofilms are covered by the extracellular matrix (ECM) secreted by bacteria. Components of the ECM include exopolysaccharides (EPS), extracellular DNA (eDNA), RNA, proteins, and lipids [9,10]. ECM protects bacterial biofilms from unfavorable environmental conditions; thus, bacteria in biofilms tend to be more resistant to various disinfectants and antimicrobial agents [11].

This resistance contributes to numerous problems associated with biofilms: limited penetration of antimicrobial substances into biofilms, differences in the metabolic activity and growth rate of individual bacterial cells, the presence in the population of cells capable of surviving under stress conditions, and the expression of undetected resistance genes [12]. In this respect, alternative antimicrobial agents active against antibiotic-resistant clinical strains of bacteria, including the *P. aeruginosa* species, bacterial viruses—bacteriophages—are of particular interest. Phage therapy has been successfully used in surgery, in the treatment of wounds, burns, and several chronic infections caused by *P. aeruginosa*, including the treatment of pulmonary and urologic infections [13,14,15]. Several studies demonstrated the effectiveness of bacteriophages for the destruction of biofilms, which opens new opportunities for treatment [16,17].

Bacteria employ two well-characterized defense systems—restriction-modification (RM) and clustered regularly interspaced short palindromic repeats with CRISPR-associated proteins (CRISPR-Cas)—to recognize and cleave phage DNA at specific genomic loci, thereby safeguarding their genomes from phages [18]. Nevertheless, phages have evolved sophisticated counterstrategies to subvert these immune mechanisms.

CRISPR-Cas systems—comprising CRISPR sites and cas genes—represent the only known adaptive and heritable bacterial immunity [19]. However, phages neutralize this defense through diverse tactics, including the production of anti-CRISPR proteins (e.g., Acr) that directly inactivate CRISPR-Cas immune complexes and evade recognition through mutations in protospacer-adjacent motifs (PAMs) or seed sequences, deletion of protospacer regions, or chemical modification of protospacer DNA, all of which impair CRISPR RNA targeting [20]. Giant phages deploy an even more sophisticated strategy by forming nucleus-like compartments that sequester phage DNA, physically blocking Cas complex assembly and preventing targeted cleavage [21].

Giant phages (Phikzvirus) have larger genomes and may encode additional depolymerases, potentially enhancing biofilm penetration. Conventional phages (Pbunavirus) are smaller and may diffuse more readily. Comparing the antibiofilm activity of these phages using optical density measurements and SEM is important for understanding the potential of phage therapy in the presence of biofilms at sites of bacterial infection. Phikzvirus and Pbunavirus are often components of therapeutic phage preparations [22]. While Phikzvirus and Pbunavirus phages have been studied individually, their comparative antibiofilm efficacy against a diverse panel of clinical *P. aeruginosa* isolates has not been systematically evaluated. We hypothesize that the phage genus influences biofilm degradation, but that strain-specific factors (e.g., EPS composition) may override genus-level trends.

The aim of this work is to evaluate the ability of giant Phikzviruses and conventional Pbunaviruses to disrupt biofilms formed by new antibiotic-resistant clinical isolates of *P. aeruginosa*, and analyze the morphology of phage interactions with clinical isolates and their biofilms.

## 2. Materials and Methods

### 2.1. Bacteriophages genera

Genus Phikzvirus: phiKZ, NN, and phiOP and another 12 phages (see Supplemental Tables S1 and S2) were isolated in the Laboratory of Bacteriophage Genetics of the I.I. Mechnikov Research Institute of Vaccines & Sera from natural sources [23,24].

Genus Pbunavirus: PB1, phi10/2, and phi14/1. Phage PB1 is from the Lindberg typing collection; it was a donation from Prof. H. Ackerman (Canada); phages SN, BP1—obtained from Dr. N.N. Sykilinda (Laboratory of Molecular Bioengineering, IBCh RAS, Moscow, Russia); E79 was obtained from Dr. B. Holloway (Australia); phages phi10/2, phi14/1, phi9/3, and phi1214 were isolated in the Laboratory of Bacteriophage Genetics of the I.I. Mechnikov Research Institute of Vaccines & Sera from natural sources [25].

### 2.2. Bacterial Strains

Standard laboratory strain *P. aeruginosa* PAO1 is a museum laboratory strain used as a host for bacteriophages and as a control strain in experiments to study the ability to form biofilms from the collection of the Laboratory of Bacteriophage Genetics at the I.I. Mechnikov Research Institute of Bacteriophage Genetics.

Clinical isolates of *P. aeruginosa* used in this work from chronic pulmonary (20 strains) and urological (20 strains) infections are from the collection of the Laboratory of Bacteriophage Genetics of the I.I. Mechnikov Research Institute of Vaccines & Sera (Received earlier from N.A. Lopatkin Research Institute of Urology, Research Institute of Pulmonology of FMBA of Russia, Museum of Bacterial Cultures of I.M. Sechenov First Moscow State Medical University, N.F. Gamaleya Institute of Microbiology and Epidemiology).

### 2.3. Antibiotic Sensitivity Testing of P. aeruginosa Clinical Isolates

The sensitivity of clinical isolates of *P. aeruginosa* to antibacterial drugs was determined by the disk-diffusion method on lawns of bacteria grown on Müller-Hinton agar, according to the EUCAST disk diffusion method [26]. A set of 11 DI-PLS-50-01 indicator disks (NICF, Saint Petersburg, Russia) was used. The degree of sensitivity was established according to manufacturer’s recommendations.

### 2.4. Biofilm Formation Ability

Biofilm formation ability of bacteria was analyzed according to the protocols described by Peretz et al. [27] and Stepanović et al. [28] with minor modifications. Briefly, 48-well plates were filled with sterile LB medium in the volume of 990 µL, followed by adding 10 µL of overnight culture of *P. aeruginosa* strains with a concentration of 10^8^ CFU/mL. LB medium and *P. aeruginosa* PAO1 (biofilm-producing strain) were used as controls. Incubation was carried out at 37 °C for 24 h. After that, the medium was carefully removed, and wells were washed for 2–3 min with sterile phosphate-buffered saline (PBS) and air-dried. Following that, wells were filled with 1% crystal violet (CV) solution (Sigma, Stockholm, Sweden) and incubated at room temperature for 10 min. After removing the dye solution and washing the wells with PBS, the remaining dye was solubilized with 95% ethanol and the optical density of the adherent biofilm was measured on a KFK-2MP photometer at a wavelength of 590 nm. For each strain, biofilm formation experiments were carried out in at least three repetitions.

For interpretation of biofilm results, the isolates were classified according to Stepanović et al. [28], based on the following optical density (OD) average values: OD(isolate) ≤ ODc = non-biofilm-producing; OD(control) ≤ OD(isolate) ≤ 2OD(control) = weak-producing; 2OD(control) ≤ OD(isolate) ≤ 4OD(control) = moderate-producing; and 4OD(control) ≤ OD(isolate) = strong-producing.

ODc was calculated as an optical density of pure LB medium + 3*SD (SD—standard deviation).

### 2.5. Determination of Clinical Isolate Phage Susceptibility

Phage susceptibility was determined using a spot-test method. Drops (5 μL) of chloroform-extracted phage suspensions were applied using a needle replicator to freshly seeded bacterial cultures of clinical isolates prepared using the two-layer method; after air-drying, the cultures were incubated for 16–18 h at 37 °C. The transparency of the lysis spot served as the criterion for assessing sensitivity.

### 2.6. Efficiency of Plating (EOP)

Tenfold serial dilutions of the phages were plated on semi-solid agar containing a bacterial strain; after overnight incubation, the number of plaque-forming units (PFU) was counted for each phage–bacteria pair. EOP was calculated as the ratio of the number of PFUs on the clinical isolate plate to the number of PFUs on the reference strain *P. aeruginosa* PAO1. The results were classified as follows: an EOP coefficient ≥ 0.5 was considered high productivity; 0.5 > EOP ratio ≥ 0.1 was considered moderate productivity; 0.1 > EOP ratio > 0.001 was considered low productivity; and an EOP ratio ≤ 0.001 was considered ineffective [29].

### 2.7. Evaluation of Bacteriophage-Induced Biofilm Degradation

Biofilms were prepared as described above, from an initial inoculum of ~10^8^ CFU/mL, but the pre-cultivation time was increased to 72 h. All biofilms were formed under identical conditions. Phage stocks were prepared fresh for each experiment. The same volume (100 µL) of phage suspension was added to each well. Incubation time was 24 h or 48 h post-phage-addition. After removal of the medium, plates were air-dried, and phage solution was added to the cells to the amount of 1 mL, achieving three different values of multiplicity of infection (MOI)—0.001, 0.01, and 0.1, based on this initial titer. For an initial bacterial density of ~10^8^ CFU/mL, MOI 0.001, 0.01, 0.1, and 1 corresponded to 10^5^, 10^6^, 10^7^, and 10^8^ PFU/mL, respectively. Additional incubation was performed for one day followed by CV staining and optical density evaluation. For each phage with a particular MOI, the experiment was performed in at least three repetitions.

### 2.8. CV Staining of Bacterial Biofilms

Overnight bacterial cultures (10 µL) were inoculated into 1 mL of fresh LB medium per well and incubated for 24 h. After incubation, the medium was carefully aspirated using a syringe to minimize disruption of the biofilm. The biofilm was then washed twice with PBS to remove planktonic cells. Biofilms were treated with different phage lysates and incubated for 24 h. Following treatment, the reaction mixture was aspirated, and the biofilms were washed again with PBS. The samples were stained with 1% (*w*/*v*) CV solution for 15 min at room temperature. Unbound dye was removed by extensively washing the wells with PBS (≥3 washes). Plates were air-dried overnight before imaging.

### 2.9. Scanning Electron Microscopy

For scanning electron microscopy (SEM), the biofilm samples before and after phage treatment were caught using aluminum foil and fixed at room temperature for 30 min with a freshly prepared solution of 2% (*v*/*v*) glutaraldehyde and 3% (*v*/*v*) paraformaldehyde in 100 mM PBS, as previously described [30]. For the dehydration, foil strips with biofilms were placed into increasing concentrations of ethanol for 10 min each, transferred to acetone and subjected to critical-point drying in Hitachi HCP-2 (Hitachi, Tokyo, Japan). The samples were sputter-coated with gold and visualized in a JEM-6380 (JEOL, Tokyo, Japan). The images were edited using the GIMP 2.12 software.

### 2.10. Statistical Analysis

All data were analyzed and graphed using GraphPad Prism 9.1.0 (GraphPad Software, San Diego, CA, USA). Biofilm clearance data are presented as mean ± SD. For each MOI level (0.001, 0.01, and 0.1), two-way ANOVA was performed with bacterial isolates (Ur1, Ur14, Lu3, Lu9) and phage types (Phikzvirus and Pbunavirus) as the two categorical factors to assess differences in the percentage of remaining or clearing biofilm, followed by Tukey’s post hoc test for multiple comparisons. The significance threshold was set at α = 0.05, and significance levels are indicated as * *p* < 0.05, ** *p* < 0.01, and *** *p* < 0.001. To quantify the biological magnitude of the observed differences, Cohen’s d values were calculated for each comparison and presented as grouped bar charts, where positive values indicate superior clearance by Pbunavirus over Phikzvirus, and negative values indicate superior clearance by phiKZ over PB1. All experiments were performed in three independent replicates (*n* = 3; for Ur14-Phikzvirus group, *n* = 6).

## 3. Results

### 3.1. Characterization of Antibiotic Sensitivity of Clinical Isolates from Chronic Infections and Their Ability to Form Biofilms

A standard set of 11 antibiotics active against *P. aeruginosa* bacteria was used to test the antibiotic sensitivity of 20 clinical isolates of *P. aeruginosa* from chronic urological (Ur) infections (Table 1).

The results showed that all tested Ur strains appeared to be multi-drug-resistant (MDR). Three strains (Ur1, Ur7, Ur13) were resistant to all antibiotics included in the kit. Four stains (Ur9, Ur11, Ur19, and Ur16) demonstrated intermediate sensitivity to two or more antibiotics. Other three strains (Ur8, Ur11, Ur14) had intermediate sensitivity to one or two antibiotics. Notably, amikacin and gentamicin were the most effective against this group of clinical isolates.

The same set of antibiotics was used to test the antibiotic sensitivity of 20 clinical isolates of *P. aeruginosa* from chronic pulmonary (Lu) infections (Table 2). Similarly, all isolated strains from the pulmonary infection clinical group appeared MDR. One strain (Lu18) was fully resistant to all the antibiotics, while seven strains (Lu3, Lu7, Lu10, Lu11, Lu13, Lu15, Lu20) showed intermediate sensitivity to antibiotics from the kit. Amikacin was still the most effective, yet gentamicin exhibited less activity against this group of clinical isolates.

**Figure 1:**
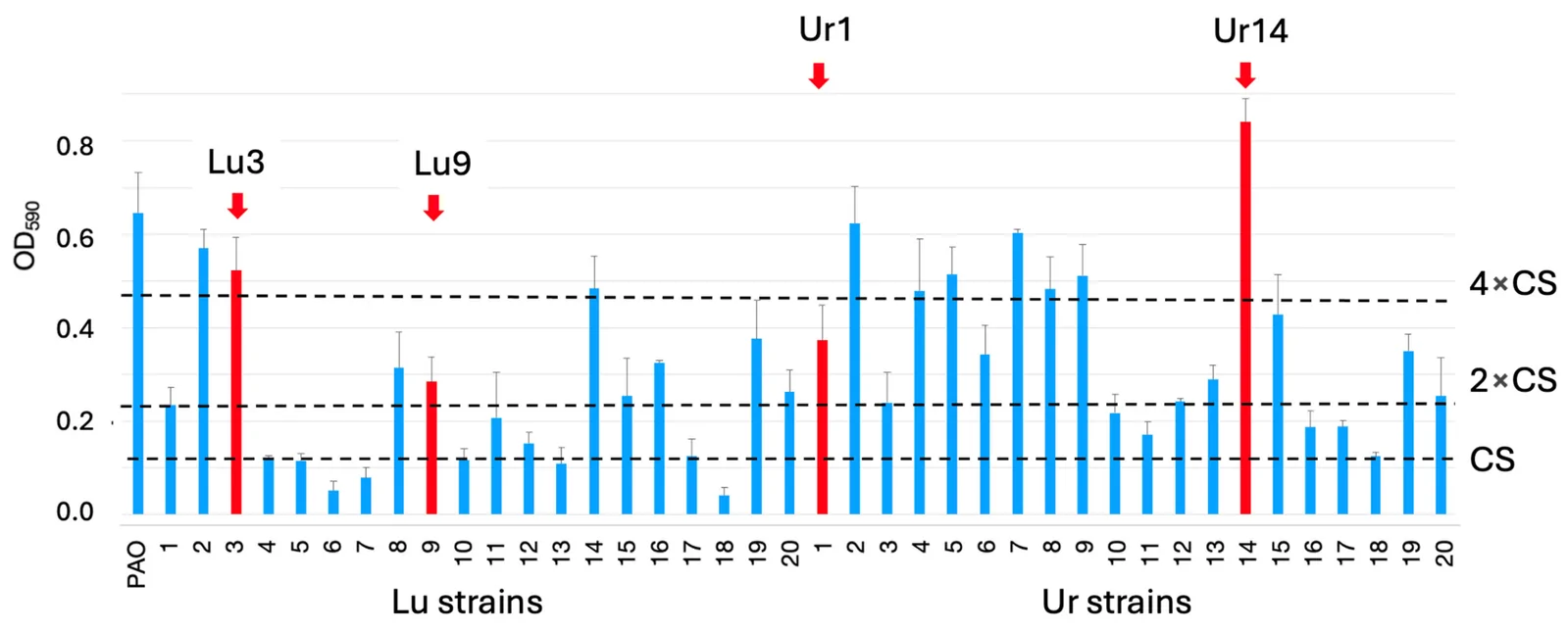
Biofilm production by *Pseudomonas aeruginosa* isolates. PAO, control; strains: # left—Lu1-Lu20, right—Ur1-Ur20, respectively. Arrows and red columns mark four strains for further phage application.

We assessed the ability of the aforementioned forty *P. aeruginosa* clinical isolates to form biofilms first by determining the optical density after CV staining (Figure 1). Most of the studied isolates can form stable biofilms. Twelve strains (Lu8, Lu9, Lu15, Lu16, Lu19, Lu20, Ur1, Ur6, Ur13, Ur15, Ur19, Ur20) exhibited moderate intensity of biofilm formation, whereas ten (Lu2, Lu3, Lu14, Ur2, Ur4, Ur5, Ur7, Ur8, Ur9, Ur14) demonstrated strong biofilm formation ability. Notably, urologic isolates included in this study had a stronger ability for robust biofilm formation. Results indicated that strains that are completely resistant to antibiotics, as well as those demonstrating partial antibiotic sensitivity, provided an equally high capacity for biofilm formation.

### 3.2. Rationale for Selecting Phages for the Study

The aim of this study is to evaluate the ability of selected bacteriophages to disrupt pre-formed *Pseudomonas aeruginosa* biofilms. Each group of *P. aeruginosa* clinical isolates was tested for susceptibility to 24 phages of the Phikzviruses and eight phages of the Pbunaviruses (Supplementary Tables S1 and S2). The most active phages against the clinical isolates under study were those of the genus Pbunavirus (eight out of 20 urological strains and nine out of 20 pulmonary strains were completely lysed) (Supplementary Tables S1 and S2). On the other hand, Phikzviruses, in most cases, did not completely lyse bacterial cultures (due to their ability to pseudolysogeny), but they exhibit a noticeable interaction with virtually all of the clinical isolates used in this study. The strains used in the study, isolated from chronic pulmonary infections, turned out to be more resistant to Phikzviruses bacteriophages. Based on the phage susceptibility test results, we selected two strains with moderate (Lu9 and Ur1) and strong (Lu3 and Ur14) biofilm formation ability; each of them undergoes lysis upon exposure to Phikzviruses (phiKZ, NN and phiOP) and Pbunaviruses (phi10/2, PB1 and phi14/1).

**Figure 2:**
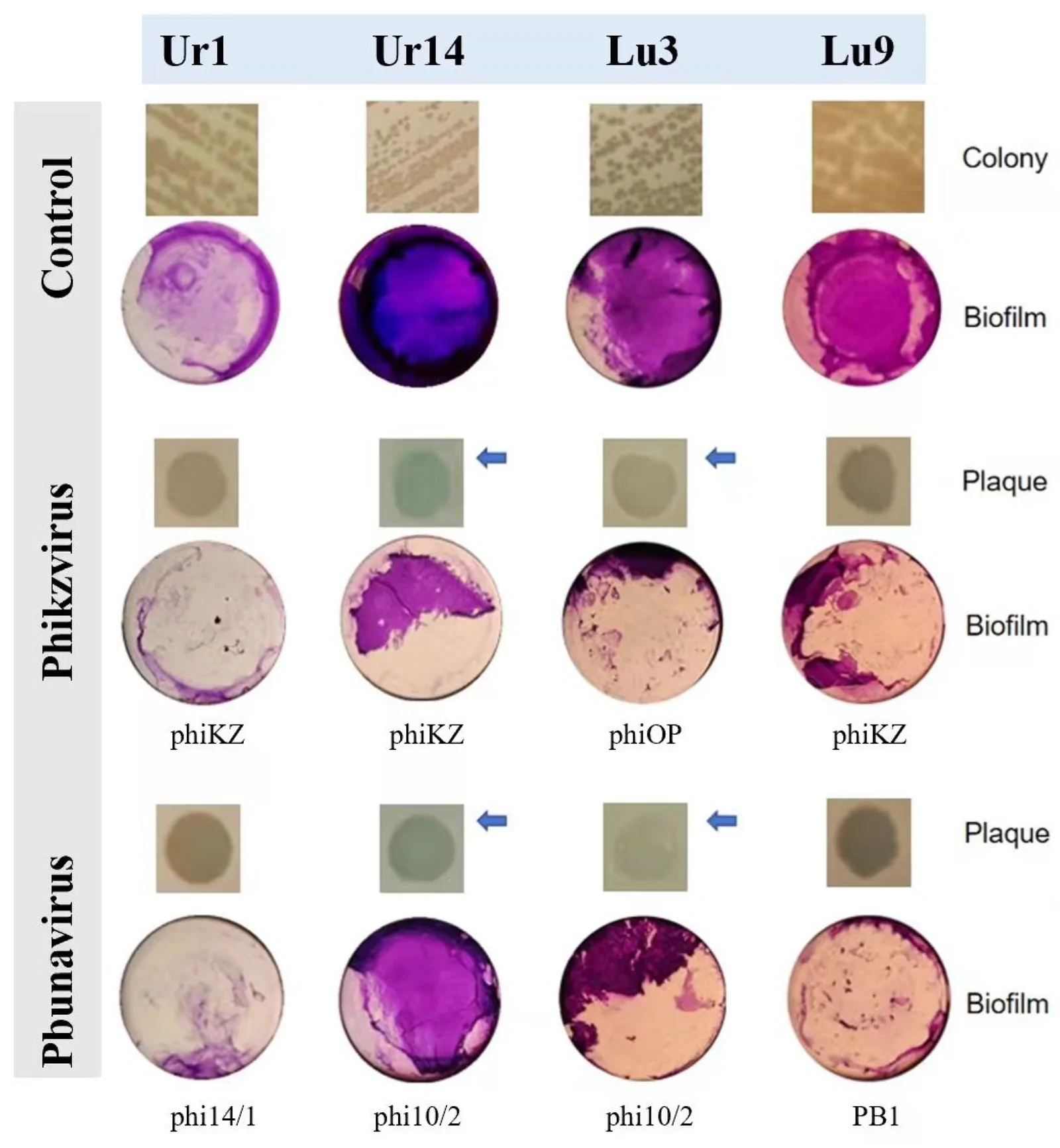
Plaque assay (top) and biofilm CV staining (bottom) of four clinical isolates (24 h biofilm growth). Arrows are pointing to green pigmentation, indicative of pyocyanin production.

Plaques formed by equivalent concentrations of phage lysates (10^8^ PFU/mL) showed comparable diameters across all susceptible strains, indicating consistent lytic efficiency under standardized conditions (Figure 2). Bacteriophage EOP was evaluated for all phages and bacterial strains used in the study. Table 3 presents the results of the efficiency of lysis (EOL) assessments based on spot-test results (explained in Supplemental Figure S1). Most phages exhibit high or moderate EOL against studied clinical isolates. EOP does not always correlate directly with the EOL, as determined by spot testing. This is particularly evident for phage phi10/2 growing on strain Ur14—extensive lysis despite low EOP. This may be because this phage, even at a relatively low titer, can completely lyse susceptible bacteria within the application zone. Similarly, no strict correlation was observed when comparing the EOP and antibiofilm activity of bacteriophages (see below). Thus, while in the case of strain Ur14 one might expect low antibiofilm activity of phages due to reduced EOP, phages effectively reduced the Ur1 biofilm even with moderate EOP.

### 3.3. Ability of Bacteriophages to Degrade Biofilms of Clinical Isolates

After overnight growth, a layer of pellicle biofilm was located on the liquid–air interface of the broth medium. Biofilms formed by strains Ur1 and Lu9 (with moderate biofilm formation ability) were characterized by high viscosity and low elastic recovery. These biofilms were thick yet could easily be deformed when external force was applied (e.g., pipette tip contact). Strains Ur14 and Lu3 biofilms (strong biofilm formation ability) exhibited low viscosity; biofilms were thinner, forming a rather rigid structure resistant to any mechanical disruption. CV staining of untreated biofilms (Figure 2, top row) also revealed substantial differences: Ur14 formed the most robust biofilms, Lu3 and Lu9 biofilms demonstrated intermediate thickness, and Ur1 produced the thinnest biofilm.

We tested the anti-biofilm effect of six selected bacteriophages against 72 h mature biofilms using OD_590_ assays. OD_590_ readings were used as a relative measure of biofilm biomass. We acknowledge that differences in EPS composition or cell packing between strains may affect absorbance; thus, results are presented as percentage reduction relative to untreated controls for each strain. The percentages of the remaining biofilm following 24 h exposure to bacteriophages at different multiplicity of infection (MOI = 0.001, 0.01 and 0.1) are presented in Table 4.

Tested bacteriophages affected biofilms with different capabilities (Table 4). Bacteriophages of both genera successfully reduced >50% of the biofilm of clinical isolates Lu3 (strong biofilm formation ability) and Lu9 (moderate ability). A very important parameter is the multiplicity of infection—MOI. Lu3 biofilm was most effectively reduced after exposure to Pbunavirus phi10/2 at MOI 0.001 (Table 4). For strain Lu9, the biofilm was effectively reduced by Phikzvirus phiKZ at MOI 0.01 (Table 4). It is noteworthy that a 10-fold change in the MOI of phage phiKZ (a 10-fold increase or decrease in phage titer) in the case of strain Lu9 resulted in a 22–26% decrease in biofilm lysis efficiency. At MOI 0.01, only 24.6% of the film remains, while at MOI 0.1, 50.7% remains and at MOI 0.001, 47.0% remains.

On the urologic isolates Ur1 (moderate) and Ur14 (strong), we tested the following phages: phiKZ and NN (Phikzvirus), phi14/1, phi10/2 and PB1 (Pbunavirus). The difference in the ability of bacteriophages to reduce biofilms formed by each of the strains (or of a specific strain) was more pronounced (Table 4, Figure 2). Ur1 biofilm was effectively degraded by both phage genera, achieving between 58% and 82% degradation rate. In contrast, only 14% to 45% of Ur14 biofilm was degraded by phages of both groups, although they have shown good efficacy to reduce biofilm biomass of other clinical isolates. The best results in Ur14 biofilm reduction were achieved with the application of NN phage (Phikzvirus) at MOI 0.1 and 54.5% of the remaining biofilm.

Typically, when phages are used to treat various infections, the treatment lasts longer than one day. We decided to assess the effect of the phages on the biofilm after a 48 h incubation period (Table 5). For pulmonary clinical isolates and phages of the Pbunaviruses genus, in all experimental conditions (except Lu3/phi10/2 at an MOI of 0.001), a decrease in biofilm abundance was observed after 48 h of incubation compared to 24 h, from 35–50% to 20–40%. This may indicate stable antibiofilm activity of these phages against the Lu9 and Lu3 strains. In the case of the phiKZ phage on the Lu9 strain, for two MOI values (0.001 and 0.1), the amount of biofilm also decreased from 48–50% to 30–40%. However, incubating strain Lu3 with the phiOP phage (Phikzviruses) for 48 h leads to a reverse increase in biofilm from 40–48% to 55–60%.

For strain Ur1, as the exposure time to bacteriophages of both types increases, a further reduction in biofilm biomass occurs at most MOI values. For the more effective phage phiKZ, this reduction can reach 16.4–17.4% of the remaining biofilm. A completely opposite pattern is observed with prolonged phage exposure to the Ur14 strain’s biofilm—in all cases, the biofilm increases in size compared to the results after 24 h of phage exposure. In the case of the phiKZ phage, when exposed to a three-day-old biofilm for 48 h, it demonstrated a complete lack of efficacy, which may indicate that the strain possesses other defense mechanisms that are more effective over longer time intervals. On the other hand, such a sharp increase in biofilm production can be explained by the pseudolysogenic mechanism inherent in this phage, which, according to some data, is capable of inducing increased mucus secretion and viscosity in infected bacteria.

### 3.4. SEM Study of the Effect of Bacteriophages on Biofilms of Clinical Isolates

Next, we used SEM to visualize and compare the morphological changes in strains with moderate-to-strong biofilm-producing ability after phage application for 2 and 24 h.

**Figure 3:**
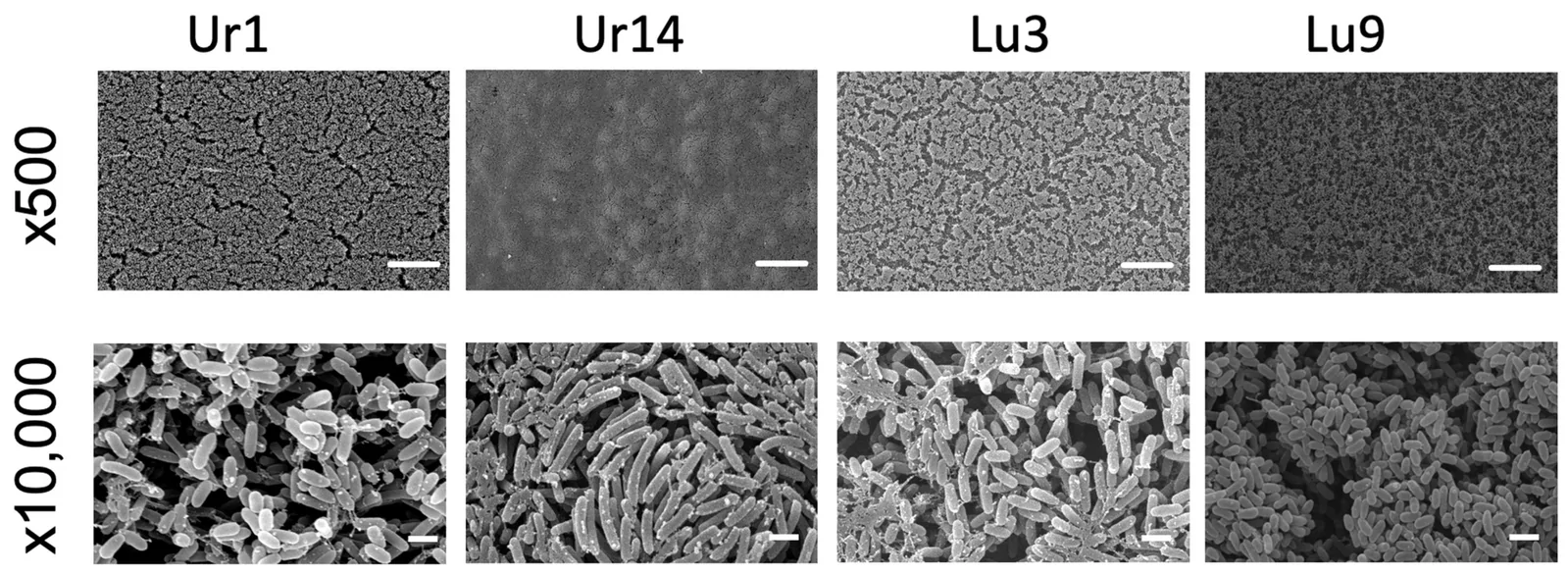
SEM of biofilms produced by *P. aeruginosa* clinical isolates Ur1, Ur14, Lu3, and Lu9. Images were taken from random regions of biofilms using magnifications (top) 500× and (bottom) 10,000×. Scale bars—10 μm and 1 μm, respectively.

Biofilms of all four strains exhibited planar architectures (Figure 3). Notably, Ur14 biofilms displayed the most compact and ordered organization, while Ur1 biofilms demonstrated significantly looser cell packing relative to other strains. This is consistent with the CV stain results (Figure 2). The few fibrous structures visible at magnification ×10,000 appear to be EPS.

**Figure 4:**
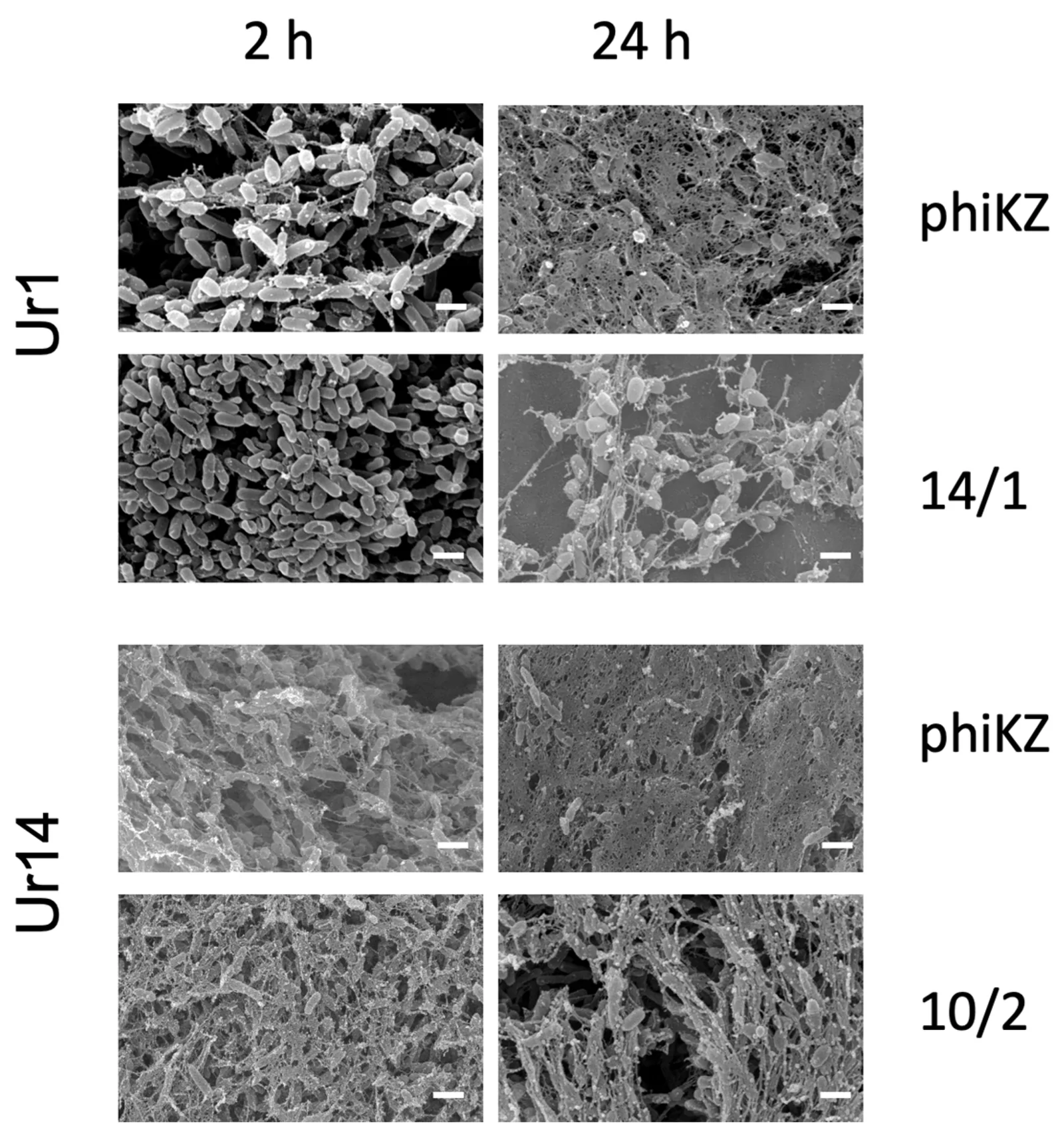
SEM visualization of phage application to biofilms produced by *P. aeruginosa* clinical isolates Ur1 and Ur14. Magnification 10,000×. Scale bars—1 μm.

**Figure 5:**
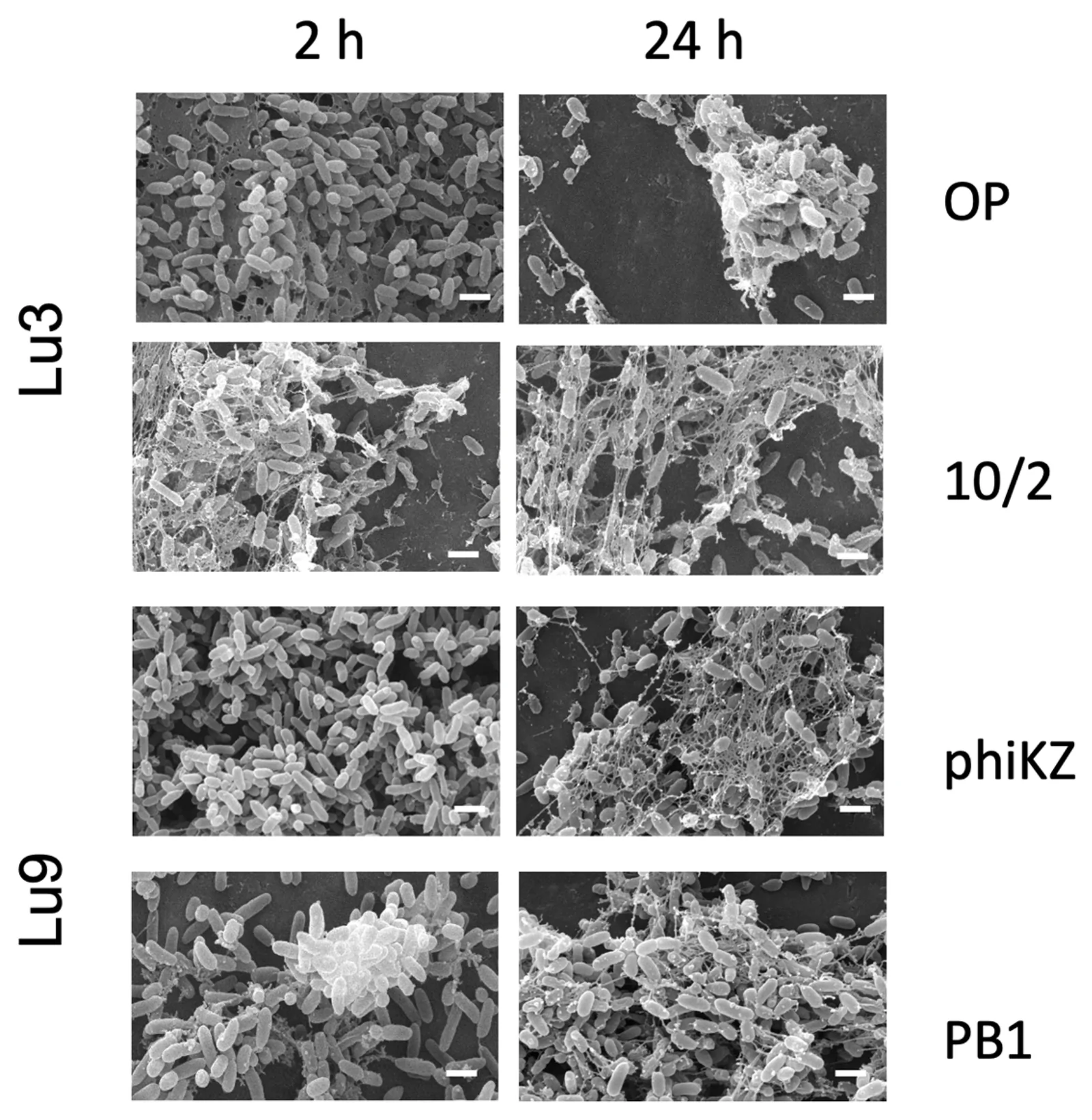
SEM visualization of phage application to biofilms produced by *P. aeruginosa* clinical isolates Lu3 and Lu9. Magnification 10,000×. Scale bars—1 μm.

Phage-mediated biofilm disruption was detectable after just 2 h of phage exposure: (Figure 4 and Figure 5). A striking feature of all phage-treated samples was the appearance of dense fibrillar networks permeating the biofilm matrix. These structures, practically absent in controls, displayed two distinct morphologies—thin, web-like filaments (e.g., Ur1) and thicker, amorphous polymers (e.g., Lu3). Fibrillar structures observed by SEM may correspond to extracellular DNA or EPS, but their chemical identity was not confirmed in this study. Exopolysaccharides and cell debris can act as a physical barrier, hindering phage movement and penetration [31]. Further in-depth research is needed to understand the specific chemical identity of these polymers. The fibrillar networks had almost covered the Lu3 and Ur14 biofilms, while Lu9 and Ur1 produced a less filamentous material. Green pigmentation (arrows on Figure 2), indicative of pyocyanin production, appeared more intense in strains Ur14 and Lu3, which may correlate with enhanced biofilm antibiotic resistance profiles [32], yet quantitative pyocyanin assays were not performed in this study.

After 24 h of phage–biofilm interaction, most of the biofilms lost their tightness and were unable to adhere to the foil strips for SEM analysis. We found large gaps in the biofilm, compared to the untreated control. Compartmentalized topology was characterized by: (i) preserved regions containing three-dimensional dense cell clusters; (ii) adjacent depleted regions showing monolayer coverage or complete cellular depletion. The overall number of intact cells decreased after exposure to phages. The most important observation was the consistent association between intact cell aggregates and dense fibrillar matrices enveloping bacterial cells. These fibrous structures may be a protective mechanism for maintaining the integrity of the biofilm structure and limiting the diffusion of phages across the physical barrier.

Ur1 biofilm exhibited significantly higher clearance than Ur14 biofilm after phage application, although both EPS densities look quite large after phage exposure to different biofilms. This paradox highlights the dissociation between structural integrity and functional biofilm deradication.

Additionally, all biofilms retained their EPS architecture after the treatment, suggesting that residual eDNA–polysaccharide complexes maintain structural cohesion even after bacterial lysis. Due to the complexity of EPS components, the degradation requires a wide range of enzymes [33]. These findings underscore that biofilm clearance efficiency depends not only on EPS disruption, but on multi-factors, such as the physical properties of the biofilm and phage lyase specificity. Moreover, the three-dimensional structure of the biofilm makes us suspect that there are still intact cells in the center or under the biofilm, which are protected by EPS and damaged cells from the surface; as a result, more time may be required for the phage to lysis.

## 4. Discussion

Bacteriophages employ unique mechanisms for disrupting bacterial biofilms that fundamentally differ from conventional antibiotics and disinfectants [34]. Phages not only effectively combat bacterial biofilms, but also efficiently target bacteria within the biofilm matrix, demonstrating versatile adaptive strategies. This provides novel insights and approaches for controlling biofilm-associated infections. Here, we compared the effect of phages from genera Phikzvirus and Pbunavirus onto pre-formed *P. aeruginosa* biofilms from clinical isolates. Our results suggest that biofilms from MDR *P. aeruginosa* clinical isolates show a heterogeneous response to phage therapy. Importantly, three of four antibiotic-resistant strains (Ur1, Lu3, Lu9) showed >50% biofilm destruction (Table 1 and Table 2), comparable with typical antibiotic efficacy against mature biofilms [35].

**Figure 6:**
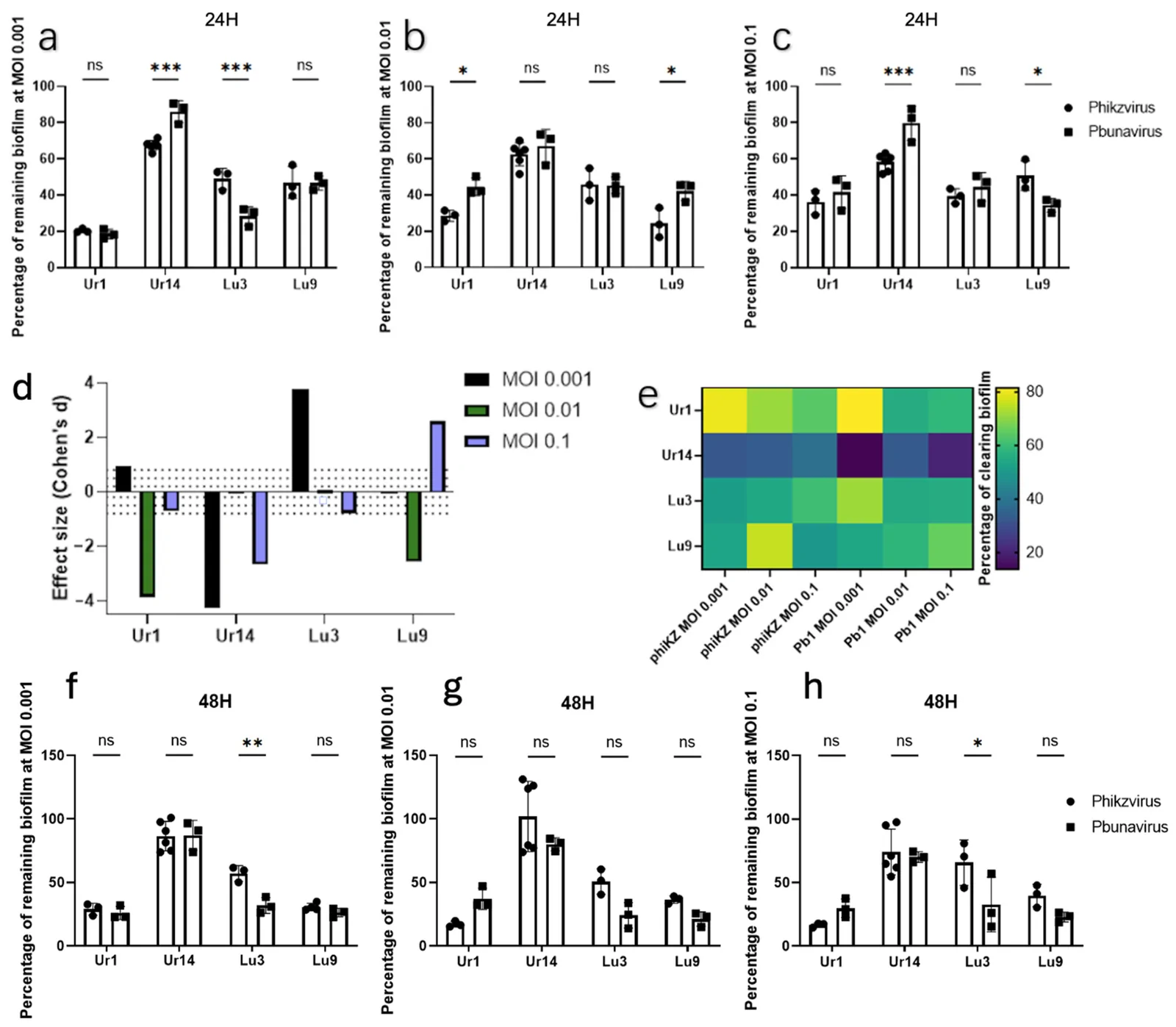
Comparison of four clinical isolates treated with Phikzviruses and Pbunaviruses at MOI 0.001 (**a**), 0.01 (**b**), and 0.1 (**c**), for 24 h; (**d**) effect sizes (Cohen’s d) for pairwise comparisons between Phikzviruses (positive values) and Pbunaviruses (negative values) across four isolates, dashed lines represent effect size thresholds: d = ±0.2 (small), ±0.5 (medium), and ±0.8 (large). (**e**) Biofilm destruction rate heatmap. Biofilm destruction rates (%) for four bacterial strains (rows) treated with Phikzviruses and Pbunaviruses at three MOI levels (six columns). Color intensity reflects the percentage of biofilm destruction. All experiments were performed in three independent replicates (*n* = 3; for Ur14-Phikzviruses, *n* = 6). Prolongation of the treatment for 48 h at MOI 0.001 (**f**), 0.01 (**g**), and 0.1 (**h**). * *p* < 0.05, ** *p* < 0.01, *** *p* < 0.001.

Bacteriophages capable of effectively lysing specific bacterial strains exhibit varying efficiencies when targeting biofilms formed by these respective strains (Figure 6). For example, phage PhiKZ can to some extent lyse all four tested strains (Table 4), but showed significant antibiofilm activity only against Lu9 and Ur1 strains. In most cases, a phage’s ability to reduce biofilm biomass depends largely on its MOI. Moreover, the most effective MOI for a phage may differ for each bacterial strain.

To evaluate the biofilm destruction of four clinical isolates (Ur1, Ur14, Lu3, and Lu9) by two phage genera (Phikzvirus and Pbunavirus) across three MOI levels (0.001, 0.01, and 0.1), we performed two-way ANOVA with Tukey’s post hoc correction on the percentage of remaining biofilm (Figure 6a–c). Some of the results revealed significant strain-dependent responses to phage treatment. Notably, Ur14 treated with Phikzvirus at MOI = 0.001 and MOI = 0.1 retained significantly less biofilm compared to Pbunavirus treatment, indicating superior clearance efficacy of Phikzvirus. Similarly, Ur1 exhibited significantly lower biofilm retention with Phikzvirus at MOI = 0.01. Conversely, Lu3 treated with Pbunavirus at MOI = 0.001 showed significantly better clearance than with Phikzvirus, suggesting distinct optimal dose combinations across strains.

Effect sizes (Cohen’s d) for Phikzvirus and Pbunavirus comparisons across three MOI levels were calculated and visualized as grouped bar charts (Figure 6d). Positive values indicate higher % of biofilm remaining for Phikzvirus, while negative values indicate higher % of biofilm remaining for Pbunavirus. For Ur1 and Lu9, the direction of effect reversed as MOI increased, suggesting that phage efficacy is highly dependent on MOI and bacterial isolates. For Ur14, Phikzvirus consistently outperformed PB1 at both low and high MOI, while for Lu3, Pbunavirus was better at the MOI 0.1 but Phikzvirus became superior at the MOI 0.001. Thus, while lytic activity (EOP/EOL) showed some correlation with biofilm reduction in certain strain–phage pairs (e.g., Lu9/phiKZ), other pairs (e.g., Ur14/phiKZ) deviated, suggesting that biofilm architecture can override lytic potential.

To provide a global overview of biofilm clearance efficacy across all experimental conditions, we generated a heatmap of clearing rates (%) for each bacterium–phage–MOI combination (Figure 6e). In this heatmap, yellow represents high clearance rates (effective phage treatment) and blue represents low clearance rates (poor clearance). It is clear to observe that Ur1 biofilm is the most sensitive one to these two spices of phages, especially at MOI = 0.001. Conversely, Ur14 is the most resistant, especially with Pbunavirus treatment, which is also consistent with the significant analysis results.

The most resistant to the phage treatment are biofilms of the Ur14 strain. This strain has a specific phenotype with intense pyocyanin secretion and extra matrix-barrier development. We hypothesize that EPS may act as a physical barrier restricting phage diffusion, consistent with the reduced efficacy observed for Ur14. The area observed was completely covered by EPS fibers after the phiKZ infection (Figure 4), which is attributed to the substantial presence of polysaccharides and a high viscosity associated with giant phages. Another possibility is that EPS may interact with the surfaces of compromised cells to shield the bacterial cells within the biofilm. Following a 24 h treatment with phiKZ, only a few damaged cells and a significant number of filamentous structures persisted on the biofilm’s surface (Figure 4). However, the remaining three-dimensional architecture of the biofilm leads us to believe that intact cells could remain within the deeper layers, safeguarded by both EPS and the damaged surface cells. It may require additional time for the phage to completely lyse the biofilm. Notably, other phage applications also showed low efficiency (Table 4 and Table 5), suggesting that the biofilm of the Ur14 strain may have broad-spectrum phage resistance properties. We used two phages from Phikzvirus—phiKZ and NN. The best MOI was 0.1, and phage NN reduce biofilm biomass better than phiKZ (Table 4 and Table 5). These results show that the lytic activity of a bacteriophage does not correlate directly with the biofilm reduction efficiency of a particular strain. Consequently, for the successful use of bacteriophages against bacterial biofilms, individual selection and future testing of bacteriophages for anti-biofilm activity against each infecting bacterial strain are crucial.

## 5. Conclusions

All studied bacteriophages were able to affect selected biofilms with different intensities, six of them were able to reduce more than 50% biofilms and two phages by 75%. In most cases, the phage’s ability to reduce biofilm biomass showed clear dependence on the multiplicity of infection (MOI). Bacteriophages capable of lysing a particular strain may act with different efficacy on the biofilm formed by that strain. Thus, the lytic activity of a bacteriophage does not correlate directly with their antibiofilm activity. The intensity of bacterial biofilm destruction is an individual property of each bacteriophage–bacteria pair. To achieve the most potent biofilm disruption, phages and their multiplicity of infections require individual selection for each bacterial strain. Such an evaluation allows us to add additional phage selection criteria for personalized phage therapy, and to consider bacteriophages as potential biofilm-control agents.

## Figures and Tables

**Table 1:** Antibiotic sensitivity of clinical isolates of *P. aeruginosa* isolated from chronic urological infections.

Strain	Amikacin	Ampycillin /Sulbactam	Gentamicin	Imipenem	Meropenem	Ticarciline/ Clavulanic Acid	Trimetoprim/ Sulfametoxazol	Cephepim	Cefoperazone	Ceftazidime	Ciprofloxacin
Ur1	-	-	-	-	-	-	-	-	-	-	**-**
Ur2	+	-	-	+/-	+	-	-	-	-	-	+
Ur3	+	-	+/-	+	+	-	-	-	-	-	-
Ur4	+	-	+	+	+/-	-	-	-	-	-	-
Ur5	+	-	+	+/-	-	-	-	-	-	-	-
Ur6	+	-	+	-	+/-	-	-	-	-	-	-
Ur7	-	-	-	-	-	-	-	-	-	-	-
Ur8	-	-	+/-	-	-	-	-	**-**	-	-	-
Ur9	+	-	+	+	+/-	-	-	-	-	-	+/-
Ur10	+	-	+	+	+	-	-	+/-	-	-	-
Ur11	-	-	+/-	-	+/-	-	-	-	-	-	-
Ur12	-	-	-	+/-	+	-	-	-	-	-	-
Ur13	-	-	-	-	-	-	-	-	-	-	-
Ur14	+/-	-	-	-	-	-	-	-	-	-	-
Ur15	+	-	+	+/-	-	-	-	-	-	-	-
Ur16	+/-	-	+	+/-	+/-	-	-	-	-	-	+
Ur17	+	-	-	+	+	-	-	-	-	-	-
Ur18	+	-	-	-	-	-	-	-	-	-	-
Ur19	+	-	+	+/-	+/-	-	-	**-**	-	-	+
Ur20	+	-	-	-	-	-	-	-	-	-	-

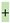
—sensitive, 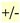
—intermediate, 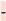
—resistant.

**Table 2:** Antibiotic sensitivity of clinical isolates of *P. aeruginosa* isolated in chronic lung infections.

Strain	Amikacin	Ampycillin /Sulbactam	Gentamicin	Imipenem	Meropenem	Ticarciline/ Clavulanic Acid	Trimetoprim/ Sulfametoxazol	Cephepim	Cefoperazone	Ceftazidime	Ciprofloxacin
Lu1	+	-	+	+/-	+/-	-	-	-	-	+/-	+/-
Lu2	+	-	+	+/-	-	-	-	-	+/-	-	-
Lu3	+/-	-	+/-	+/-	-	-	-	-	+/-	-	+/-
Lu 4	+	-	+	-	-	-	+/-	-	+/-	+/-	+/-
Lu 5	+/-	-	+/-	+/-	+	+	-	-	-	-	-
Lu 6	+/-	-	+/-	-	-	+	-	-	-	-	-
Lu 7	+/-	-	+/-	+/-	+/-	-	+/-	-	-	-	+/-
Lu 8	+	-	+/-	-	+	-	-	**+/-**	-	-	+
Lu 9	+	-	+	+/-	-	-	+/-	-	-	-	-
Lu 10	-	-	-	-	-	-	-	-	-	-	+/-
Lu 11	+/-	-	-	-	-	-	-	-	-	-	-
Lu 12	+	-	-	-	-	-	+/-	-	-	-	+/-
Lu 13	-	-	-	-	-	-	-	-	-	-	+/-
Lu 14	+	-	-	-	-	-	-	-	-	-	-
Lu 15	+/-	-	+/-	-	-	-	-	-	-	-	-
Lu 16	+	-	+	-	-	-	+	-	-	-	+/-
Lu 17	+	-	+/-	-	+/-	-	+	+/-	-	-	+/-
Lu 18	-	-	-	-	-	-	-	-	-	-	-
Lu 19	+	-	+	-	-	-	-	-	-	-	+/-
Lu 20	+/-	-	-	-	-	-	+/-	-	-	-	+/-

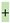
—sensitive, 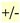
—intermediate, 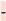
—resistant.

**Table 3:** Evaluation of bacteriophages efficiency of plating (EOP) and efficiency of lysis (EOL).

Bacterial Strain	Phikzviruses						Pbunaviruses					
	phiOP		phiKZ		NN		phi10/2		phi14/1		PB1	
	EOP	EOL	EOP	EOL	EOP	EOL	EOP	EOL	EOP	EOL	EOP	EOL
Lu 3	0.1	++					**1.52**	+++	**0.56**	+++		
Lu 9			**2.39**	++							0.38	+++
Ur 1			0.22	+++					0.24	+++		
Ur 14			0.19	+++	0.16	+++	*0.01*	+++				

Highly productive “bacterial strain-phage” pairs are marked with bold, moderately productive—underscored, and low-efficiency—italic. +++—complete lysis; ++—partial lysis. The bacteria are susceptible, but some have demonstrated resistance.

**Table 4:** Efficiency of phage therapy application (% of 72 h biofilm remaining after 24 h phage treatment).

Strain	Phikzviruses			Pbunaviruses		
	MOI			MOI		
	0.001	0.01	0.1	0.001	0.01	0.1
Lu3	phiOP			phi10/2		
%	49.1 ± 5.7	45.9 ± 8.9	39.5 ± 4.1	28.4 ± 5.2	45.5 ± 4.6	44.5 ± 7.9
Lu9	phiKZ			PB1		
%	47.0 ± 8.8	24.6 ± 8.2	50.7 ± 8.2	46.7 ± 3.8	42.3 ± 5.2	34.3 ± 3.7
Ur1	phiKZ			phi14/1		
%	20.4 ± 1.1	28.7 ± 3.0	36.1 ± 6.5	18.5 ± 2.7	42.2 ± 7.9	41.7 ± 9.0
Ur14	phiKZ			phi10/2		
%	67.8 ± 0.5	66.4 ± 3.4	61.7 ± 1.3	86.1 ± 6.0	67.1 ± 9.2	79.8 ± 9.5
Ur14	NN					
%	67.3 ± 4.3	63.6 ± 3.7	54.5 ± 3.7			

**Table 5:** Efficiency of phage therapy application (% of 72 h biofilm remaining after 48 h phage treatment).

Strain	Phikzviruses			Pbunaviruses		
	MOI			MOI		
	0.001	0.01	0.1	0.001	0.01	0.1
Lu3	phiOP			phi10/2		
%	57.1 ± 6.2	50.7 ± 10.0	65.8 ± 17.9	31.8 ± 6.2	24.1 ± 10.0	20.8 ± 7.6
Lu9	phiKZ			PB1		
%	30.6 ± 2.9	36.5 ± 3.0	39.9 ± 8.8	26.3 ± 3.4	21.2 ± 5.6	22.9 ± 3.9
Ur1	phiKZ			phi14/1		
%	28.9 ± 4.6	17.1 ± 2.3	16.6 ± 1.7	25.9 ± 5.2	37.2 ± 8.4	29.9 ± 7.3
Ur14	phiKZ			phi10/2		
%	88.4 ± 13.7	100.0 ± 14.5	85.9 ± 18.3	87.1 ± 11.7	80.0 ± 5.1	70.1 ± 4.2
Ur14	NN					
%	82.3 ± 7.8	76.8 ± 2.8	62.5 ± 8.1			

## Data Availability

Detailed protocols and results supporting the findings of this study are available within the paper. All strains of bacteria and bacteriophages used in this work are stored in the collection of the Laboratory of Bacteriophage Genetics Mechnikov Research Institute of Vaccines & Sera and are available on request from the corresponding authors. The SEM images can be found at https://pan.baidu.com (accessed on 1 August 2026) on request.

## Supplementary Materials

The following supporting information can be downloaded at: https://www.mdpi.com/article/10.3390/v18080869/s1, Table S1: Growth of the Phikzviruses and Pbunaviruses on *P. aeruginosa* strains of urological infections; Table S2: Growth of the Phikzviruses and Pbunaviruses on clinical isolates of *P. aeruginosa* pulmonary infections; Figure S1: Efficiency of lysis (EOL) assessments based on spot-test results.

## Abbreviations

The following abbreviations are used in this manuscript:

ECM	extracellular matrix
EPS	exopolysaccharides
PBS	phosphate-buffered saline
CV	crystal violet
OD	optical density
MOI	multiplicity of infection
SEM	scanning electron microscopy
EOP	efficiency of plating
PFU	plaque-forming units
MDR	multi-drug resistant
EOL	efficiency of lysis
