# Supplementary material for: The Efficacy of Giant Phikz Viruses in Degrading Biofilms of Pseudomonas aeruginosa in Comparison to Pbuna Viruses
Source: Viruses. 2026 Aug 9;18(8):869. doi: 10.3390/v18080869 (PMC13517294; doi:10.3390/v18080869)
Supplement: Supplementary file 1 [file viruses-18-00869-s001.zip › viruses-4450526-supplementary.pdf]

Supplemental Table S1. Growth of the Phikzviruses and Pbunaviruses on *P. aeruginosa* strains of urological infections.

| Phages | Clinical isolates of *P. aeruginosa* | | | | | | | | | | | | | | | | | | | |
|---|---|---|---|---|---|---|---|---|---|---|---|---|---|---|---|---|---|---|---|---|
| | 1 | 2 | 3 | 4 | 5 | 6 | 7 | 8 | 9 | 10 | 11 | 12 | 13 | 14 | 15 | 16 | 17 | 18 | 19 | 20 |
| Phikzviruses | | | | | | | | | | | | | | | | | | | | |
| phiKZ | +++ | ++ | + | + | +/- | ++ | + | + | + | ++ | + | + | + | +++ | + | ++ | +/- | +/- | ++ | +/- |
| NN | +++ | ++ | + | + | - | ++ | + | + | + | + | +/- | + | + | +++ | +/- | ++ | - | +/- | ++ | +/- |
| LBG 20 | +++ | ++ | +/- | +/- | - | + | + | + | + | + | + | + | + | ++ | + | + | - | - | ++ | +/- |
| LBG 21 | +++ | ++ | +/- | +/- | - | + | + | + | + | + | + | + | + | ++ | ++ | + | - | - | ++ | +/- |
| LBG 23 | +++ | ++ | + | + | - | + | + | + | + | + | + | + | + | ++ | + | + | - | - | ++ | - |
| LBG 25 | +++ | ++ | +/- | +/- | - | + | + | +/- | +/- | ++ | - | +/- | + | + | + | ++ | + | +/- | ++ | - |
| LBG 26 | +++ | ++ | + | + | - | + | + | + | + | + | + | + | + | ++ | + | ++ | - | + | ++ | +/- |
| PBD 1 | +++ | ++ | + | + | - | + | + | + | + | + | +/- | + | + | ++ | + | + | - | - | ++ | +/- |
| PBD 2 | +++ | ++ | + | + | - | + | + | + | + | + | + | + | + | + | +/- | ++ | - | +/- | ++ | +/- |
| PBD 3 | +++ | ++ | + | + | - | + | + | + | + | + | +/- | + | + | ++ | + | ++ | +/- | +/- | ++ | +/- |
| PBD4 | +++ | ++ | + | + | - | + | + | + | + | ++ | + | + | + | ++ | + | + | - | +/- | ++ | +/- |
| ϕKZ 3 | +++ | ++ | + | + | - | + | + | + | + | + | + | + | +++ | ++ | ++ | + | - | +/- | ++ | - |
| ϕKZ 10 | +++ | ++ | + | + | +/- | ++ | + | + | + | + | ++ | + | + | +++ | + | + | - | +/- | ++ | +/- |
| OП | +++ | ++ | + | + | + | ++ | + | + | + | + | ++ | +++ | + | ++ | + | + | - | +/- | ++ | +/- |
| Ser | ++ | ++ | + | + | - | + | + | + | + | + | + | + | + | ++ | ++ | ++ | - | +/- | ++ | +/- |
| Pbunaviruses | | | | | | | | | | | | | | | | | | | | |
| PB1 | - | ++ | +++ | ++ | ++ | ++ | ++ | +/- | - | - | +/- | - | - | +/- | - | - | - | - | ++ | - |
| 10/2 | +++ | ++ | +++ | ++ | +++ | ++ | ++ | - | - | +++ | +/- | - | ++ | +++ | ++ | +/- | - | + | +++ | + |
| SN | +++ | ++ | +/- | - | +++ | +++ | ++ | - | - | +++ | +/- | - | ++ | + | ++ | + | - | + | ++ | + |
| 1214 | +++ | ++ | +/- | + | +++ | +++ | ++ | - | + | +++ | +/- | + | ++ | - | +++ | + | - | ++ | +++ | + |
| BP1 | - | ++ | +++ | ++ | +++ | ++ | ++ | - | - | - | +/- | - | - | + | - | + | - | +/- | +++ | +/- |
| E79 | - | ++ | +++ | ++ | +++ | ++ | ++ | - | - | - | +/- | - | - | + | - | +/- | - | - | +++ | - |
| 14/1 | +++ | ++ | - | - | +++ | +++ | ++ | - | - | +++ | +/- | +/- | ++ | - | +++ | +/- | - | + | ++ | - |
| 9/3 | - | ++ | +++ | +++ | +++ | +++ | ++ | - | - | - | +/- | - | - | ++ | - | + | - | + | +++ | + |

Note: +++ - complete lysis; ++ - partial lysis; + - weak lysis; +/- - phage-bacteria interaction present; - - complete absence of lysis and interaction. Strains numbered 1-20 in the table represent the strains Ur1-Ur20

Supplemental Table S2. Growth of the Phikzviruses and Pbunaviruses on clinical isolates of *P. aeruginosa* pulmonary infections

| Phages | Clinical isolates of P. aeruginosa | | | | | | | | | | | | | | | | | | | |
|---|---|---|---|---|---|---|---|---|---|---|---|---|---|---|---|---|---|---|---|---|
| | 1 | 2 | 3 | 4 | 5 | 6 | 7 | 8 | 9 | 10 | 11 | 12 | 13 | 14 | 15 | 16 | 17 | 18 | 19 | 20 |
| **Phikzviruses** | | | | | | | | | | | | | | | | | | | | |
| phiKZ | ++ | + | +/- | + | +/- | + | + | + | ++ | ++ | +/- | ++ | + | +/- | + | ++ | + | + | - | +++ |
| NN | ++ | + | - | +/- | - | | + | - | - | ++ | +/- | ++ | + | +/- | + | ++ | + | +/- | - | +++ |
| LBG 20 | ++ | +/- | + | +/- | - | - | - | +/- | - | + | - | +/- | - | - | - | - | +/- | - | +/- | +/- |
| LBG 21 | + | +/- | + | +/- | - | - | - | +/- | - | ++ | - | + | - | - | - | - | - | - | - | +/- |
| LBG 23 | ++ | +/- | + | + | - | - | +/- | +/- | - | ++ | - | + | - | - | - | - | - | - | - | +/- |
| LBG 25 | ++ | +/- | + | +/- | - | ++ | - | +/- | ++ | ++ | - | + | - | - | - | + | - | - | - | +/- |
| LBG 26 | + | ++ | ++ | +/- | +/- | +/- | +/- | +/- | - | ++ | - | - | - | - | +/- | +/- | +/- | +/- | +/- | +/- |
| PBD 1 | + | + | + | +/- | +/- | +/- | +/- | - | - | + | - | +/- | +/- | - | +/- | +/- | +/- | +/- | +/- | +/- |
| PBD 2 | + | + | +/- | +/- | +/- | +/- | +/- | +/- | - | + | + | +/- | - | - | +/- | +/- | +/- | +/- | +/- | +/- |
| PBD 3 | + | + | + | +/- | +/- | +/- | - | +/- | - | + | +/- | +/- | +/- | - | +/- | - | +/- | +/- | +/- | + |
| PBD4 | + | +/- | +/- | +/- | +/- | +/- | +/- | +/- | - | ++ | +/- | - | - | - | - | - | - | - | - | + |
| ϕKZ 3 | ++ | + | + | +/- | +/- | + | + | + | - | ++ | +/- | + | +/- | - | +/- | - | +/- | +/- | +/- | ++ |
| ϕKZ 10 | ++ | + | ++ | ++ | +/- | + | +/- | + | + | + | +/- | +/- | +/- | - | +/- | + | + | +/- | - | + |
| OП | + | +/- | ++ | + | - | +/- | +/- | +/- | +/- | ++ | +/- | +/- | +/- | - | +/- | - | - | +/- | +/- | + |
| Ser | ++ | +/- | +/- | + | - | +/- | +/- | ++ | + | ++ | - | +/- | + | ++ | + | + | + | + | ++ | + |
| **Pbunaviruses** | | | | | | | | | | | | | | | | | | | | |
| PB1 | ++ | +++ | +++ | +++ | +++ | +++ | +++ | +++ | +++ | +/- | - | +/- | + | - | + | ++ | + | + | + | ++ |
| 10/2 | ++ | +++ | +++ | +++ | +++ | +++ | +++ | +++ | +++ | +/- | - | +/- | + | +/- | + | +++ | + | + | +/- | ++ |
| SN | ++ | +++ | +++ | +++ | +++ | +++ | +++ | +++ | +++ | +/- | +/- | +/- | + | +/- | + | ++ | + | + | ++ | ++ |
| 1214 | ++ | +++ | +++ | +++ | +++ | +++ | +++ | +++ | +++ | +/- | +/- | +/- | + | +/- | + | ++ | + | + | +/- | ++ |
| BP1 | ++ | +++ | +++ | +++ | +++ | +++ | +++ | +++ | +++ | +/- | - | +/- | + | +/- | + | ++ | + | + | - | ++ |
| E79 | ++ | +++ | +++ | +++ | +++ | +++ | +++ | +++ | +++ | +/- | - | +/- | + | +/- | + | ++ | + | + | - | ++ |
| 14/1 | ++ | +++ | +++ | +++ | +++ | +++ | +++ | +++ | +++ | +/- | - | +/- | + | +/- | + | +++ | + | + | - | ++ |
| 9/3 | ++ | +++ | +++ | +++ | +++ | +++ | +++ | +++ | +++ | +/- | +/- | +/- | + | +/- | + | ++ | + | + | - | ++ |

Note: +++ - complete lysis; ++ - partial lysis; + - weak lysis; +/- - phage-bacteria interaction present; - - complete absence of lysis and interaction. Strains numbered 1-20 in the table represent the strains Lu1-Lu20

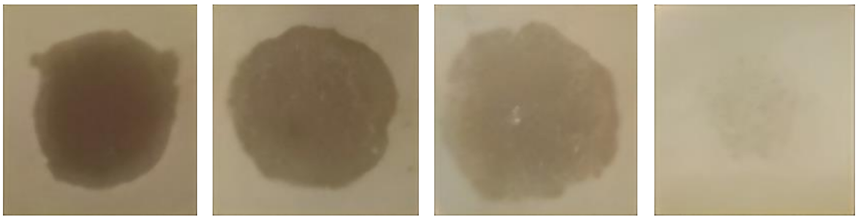

+++ ++ + +/-

complete lysis partial lysis weak lysis some interaction is present

Supplemental Figure S1. Efficiency of lysis (EOL) assessments based on spot-test results.
